# Differentiation of deer tendons from cattle tendons by a loop-mediated isothermal amplification (LAMP) test and bone remodeling bioassays

**DOI:** 10.1186/s13020-015-0065-6

**Published:** 2015-11-12

**Authors:** Li-Li Jiang, Cheuk-Lun Liu, Yuk-Lau Wong, Chun-Fong Nip, Pang-Chui Shaw

**Affiliations:** State Key Laboratory of Phytochemistry and Plant Resources in West China (CUHK), The Chinese University of Hong Kong, Shatin, N.T., Hong Kong, China; Li Dak Sum Yip Yio Chin R & D Centre for Chinese Medicine, The Chinese University of Hong Kong, Shatin, N.T., Hong Kong, China; School of Life Sciences, The Chinese University of Hong Kong, Shatin, N.T., Hong Kong, China

## Abstract

**Background:**

Deer tendons are believed more effective than cattle tendons in tonifying *kidney yang* (*shen yang*) and enhancing bone and tendons. This study aims to differentiate the two types of tendons by a loop-mediated isothermal amplification (LAMP) test and bone remodeling bioassays.

**Methods:**

Internal control primers to detect both types of tendons and specific primers for deer tendons were designed according to a sequence analysis. The LAMP test was set up and the results were analyzed by conventional gel electrophoresis, real-time fluorescence observation, and colorimetric detection. Crude tendon extracts were prepared by water extraction to compare their effects on bone. The anti-osteoclastic effects were investigated on mouse pre-osteoclast Raw264.7 cells by cell viability determination and tartrate-resistant acid phosphatase staining. The osteogenic effects were examined using rat osteoblast-like UMR106 cells by evaluation of cell proliferation, alkaline phosphatase activity, and calcium deposition. The relative gene expressions of bone remodeling-related markers, including nuclear factor of activated T-cells cytoplasmic 1, tartrate-resistant acid phosphatase, cathepsin K, and osteoprotegerin/receptor activator of NF-κB ligand, were determined by real-time PCR.

**Results:**

In the LAMP test, both deer and cattle tendons were detected in the control reactions, while only deer tendons were amplified by the specific LAMP test. In the bioassays, both tendons inhibited the viability and differentiation of pre-osteoclast Raw264.7 cells, and promoted the proliferation and mineralization of osteoblast-like UMR106 cells. The mRNA expressions of bone remodeling-related markers were consistent with the results of the bioassays.

**Conclusion:**

This study demonstrated that the isothermal LAMP test can distinguish between deer tendons and cattle tendons. Both types of tendons exhibited similar beneficial effects on bone remodeling according to the bioassay findings.

**Electronic supplementary material:**

The online version of this article (doi:10.1186/s13020-015-0065-6) contains supplementary material, which is available to authorized users.

## Background

In traditional Chinese medicine, deer tendons are the dried limb tendons of sika deer (*Cervus nippon*) or red deer (*Cervus elaphus*), while cattle tendons are the dried tendons of domestic cattle (*Bos taurus*). In East Asia, deer tendons are believed to enhance physical and sexual functioning in humans, by tonifying *kidney yang* (*shen yang*) and strengthening bone and tendons [1]. The effects of deer tendons on osteoporosis and bone loss have been studied in rat models [2, 3]. Cattle tendons are commonly used as a less effective substitute. Owing to the limited supply and high demand, the price of genuine deer tendons is about five to ten times higher than that of cattle tendons [4]. This leads to frequent substitution of deer tendons with cattle tendons by dishonest sellers. In September 2011, the Hong Kong Customs and Excise Department seized about 112 kg of suspected fake deer tendons worth about HK$41200 (US$5316) [5]. In March 2012, the same department tested the deer tendons sampled from 29 shops, and found that the samples from 28 shops were cattle tendons [6].

The two types of tendons are generally differentiated by physical examination, i.e., deer tendons are usually smaller than cattle tendons and have a lighter color [5, 6]. The Hong Kong Government Laboratory developed a deer-specific PCR test to distinguish deer tendons from cattle tendons to prevent commercial fraud [7].

Loop-mediated isothermal amplification (LAMP) is a novel isothermal amplification technique that employs a DNA polymerase with strand-displacement activity together with a set of typically four primers recognizing six specific loci on the target DNA. The reaction and detection can be finished in one step owing to the large amounts of DNA and side products produced during the reaction. The technique has been employed to detect various pathogens in the field since its first report in 2000 [8]. Recently, our group has applied LAMP to differentiate an herbal tea ingredient, *Hedyotis diffusa* Willd, from its common adulterant [9].

In the present study we developed a LAMP test to differentiate deer tendons from cattle tendons to facilitate on-site identification. Currently, the available drugs for treating bone loss and osteoporosis are mainly antiresorptive agents [10]. Some drugs like bisphosphonates also decrease bone formation [11]. Long-term use of these drugs has been associated with several side effects [10, 11]. Because both types of tendons have been used to protect bone and tendons, we evaluated the effects of extracts from the two tendon types on bone formation and bone resorption using osteoblast-like UMR106 cells and pre-osteoclast Raw264.7 cells, respectively. Both cell types are well-established models for evaluating the bone-protective effects of potential drugs [12, 13].

## Methods

### Reagents and chemicals

Reagents for cell culture, including medium, antibiotics, and serum, were purchased from Gibco, Thermo Fisher Scientific (Grand Island, NY, USA). All other chemicals were purchased from Sigma-Aldrich (St. Louis, MO, USA) unless otherwise indicated.

### Samples

**Fig. 1:**
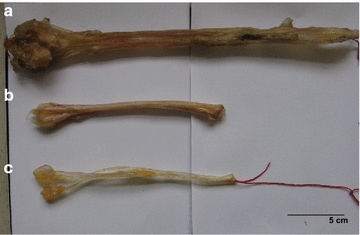
Tendons of cattle, deer and an adulterant. **a** Cattle tendon; **b** deer tendon; **c** fake deer tendon

The samples used in this study were purchased from different stores in Guangzhou (China) and Hong Kong (China). The samples were authenticated according to their morphological characteristics and confirmed by sequencing of the mitochondrial 16S rRNA gene [14]. All tested samples were kept at the Institute of Chinese Medicine, The Chinese University of Hong Kong. Detailed information and representative photos of the tested samples are shown in Additional file 1: Table S1 and Fig. 1, respectively. Deer and cattle tendon extracts were prepared from authenticated samples purchased from Hong Kong.

### DNA extraction and quantification

The tendon samples (approximately 20 mg) were cut into small pieces, and the DNA was extracted using a CTAB method [15]. The isolated DNA was quantified by a spectrophotometer (NanoDrop, Thermo Fisher Scientific, Wilmington, DE, USA) and stored at −20 °C.

### Primer design, LAMP reaction, and product detection

The DNA sequences of the 12S rRNA regions of *C. elaphus*, *C. nippon*, and *B. taurus* were retrieved from GenBank (http://www.ncbi.nlm.nih.gov/). Four common primers (F3, B3, FIP, and BIP) for the internal control reaction were designed according to the conserved regions in the three species using PrimerExplorer V4 software (http://primerexplorer.jp/e/) from Eiken Chemical Co. Ltd. (Tokyo, Japan). The deer-specific primers (F3_S, FIP_S, and BIP_S) were designed by the same software according to the polymorphic sites for differentiation in specific LAMP reactions. The sequences of the primers and their applications in the LAMP test are shown in Table 1.

**Table 1:** Information for LAMP primers

Primer	Sequence (5′ to 3′ direction)	Internal control	Specific LAMP
F3	TTTGGTCCCAGCCTTCCT	√	
B3	GCGGCTGGCACGA	√	√
FIP	TGTGTGCTTGATACCAGCTCCTCTAGCATCCACACCCCAGTGA	√	
BIP	CCTTGCATAGCCACACCCCCTCAAACTTTCGTTTATGGCTTAATTTTT	√	
F3-S	CAGCCTTCCTATTGACC		√
FIP-S	GGATGTGTGCTTGATACCAGCTCCGCATCCACACCCCAGTGAA		√
BIP-S	ACCTTGCATAGCCACACCCCCAAACTTTCGTTTATGGCTTAATTTTTA		√

The LAMP reaction was performed using an Isothermal MasterMix Amplification Kit (OptiGene, Horsham, UK) and set up in accordance with the manufacturer’s instructions. The mixture was incubated at 65 °C for 1 h in a PCR machine or portable Genie II LAMP Detector (OptiGene) followed by inactivation at 98 °C for 5 min. Three strategies were employed to detect the amplification: (1) real-time monitoring in the Genie II Detector using the fluorescent DNA binding dye provided in the kit; (2) post-reaction analysis by 1 % TAE gel electrophoresis; and (3) colorimetric detection under a UV lamp by staining with GelRed (Biotium, Hayward, CA, USA). The sensitivity of the LAMP test was determined by tenfold dilution of a DNA sample from deer tendon (500 ng/µL).

### Preparation of tendon extracts

Tendon samples were preswollen in distilled water for 48–72 h at 4 °C, and then processed to small pieces using scissors and a blender. The tendon mixtures were digested with 1:1000 (g/g) porcine pepsin (Genview, League City, TX, USA) at 37 °C for 24 h and sonicated for 15 min, before being centrifuged at 2460×*g* in a J6-MI centrifuge (Beckman Coulter Brea, CA, USA) for 30 min. The supernatants were lyophilized and stored at −20 °C.

### Cell culture

Mouse pre-osteoclast Raw264.7 cells and rat osteoblast-like UMR106 cells were obtained from ATCC (Manassas, VA, USA). All cells were cultured in Dulbecco’s modified Eagle’s medium (DMEM) supplemented with 10 % fetal bovine serum (FBS) and 100 U/mL of penicillin/streptomycin at 37 °C under 5 % CO_2_.

### Cytotoxicity and tartrate-resistant acid phosphatase (TRAP) staining in Raw264.7 cells

Raw264.7 cells were seeded in 96-well plates at 2000 cells/well and cultured overnight to allow cell attachment. The cells were further cultured with DMEM supplemented with 10 % FBS and different concentrations of tendon extracts for 4 days to determine the cytotoxicity of the tendon extracts. The medium was refreshed on day 3 (post-treatment; described below). The cytotoxicity was determined by the MTT assay as previously described [16]. For TRAP staining, the culture medium was changed to α-MEM supplemented with 10 % FBS, 80 ng/mL of receptor activator of NF-κB ligand (RANKL) (R&D Systems, Minneapolis, MN, USA), and 100 µg/mL of tendon extract on the day after cell seeding. TRAP staining was performed on day 4 using an Acid Phosphatase Kit (Sigma-Aldrich) in accordance with the manufacturer’s instructions. Osteoclasts were determined under an inverted research IX71 microscope, (Olympus, Tokyo, Japan) by TRAP-positive staining and presence of multinucleated cells (≥3 nuclei).

### Proliferation, alkaline phosphatase (ALP) activity, and calcium deposition in UMR106 cells

UMR106 cells were seeded in 96-well plates at 3,000 cells/well and cultured overnight. The culture medium was then changed to DMEM supplemented with 1 % FBS and various concentrations of the tendon extracts. DMEM containing 10 % FBS was used as a positive control. The cell proliferation was determined after 72 h of treatment using the MTT assay as described above.

UMR106 cells were seeded in 24-well plates at 10,000 cells/well and cultured overnight. The culture medium was then replaced with differentiation medium (DMEM supplemented with 10 % FBS, 50 µg/mL of ascorbic acid, and 10 mM β-glycerophosphate) containing 200–400 µg/mL of tendon extract. Cells treated with dexamethasone at 100 µM were used as a positive control. The medium was refreshed on day 3. The ALP activity was determined on day 5 using a LabAssay ALP Kit (Wako Pure Chemical Industries, Osaka, Japan) in accordance with the manufacturer’s instructions. The results were normalized by the protein contents determined using a Pierce BCA Protein Assay Kit (Pierce Biotechnology, Thermo Fisher Scientific Rockford, IL, USA).

Alizarin Red S staining was performed to detect calcium deposition. UMR106 cells were seeded in 24-well plates at 2000 cells/well and cultured overnight. The cells were treated as described for the ALP assay. On day 6, the cells were fixed with 75 % ethanol for 1 h and then stained with 40 mM Alizarin Red S for 10 min. The stain was dissolved in 10 % cetylpyridinium chloride and quantified by the absorbance at 562 nm measured in a microplate spectrophotometer (BioTek, Thermo Fisher ScientificWinooski, VT, USA).

### Real-time PCR

Raw264.7 and UMR106 cells were seeded and treated as described for the TRAP staining and ALP activity evaluations, respectively. Total RNA was extracted using TRIzol reagent (Life Technologies Carlsbad, CA, USA) as previously described [17] and quantified by the NanoDrop spectrophotometer. Total RNA (2 µg) was reverse-transcribed into cDNA by MuMLTable S2V reverse Transcriptase (Promega, Madison, WI, USA). The primers for evaluation of the relative gene expressions are shown in Additional file 1: Table S2. Real-time PCR was performed in an ABI 7500 PCR System (Applied Biosystems, Thermo Fisher Scientific, Foster City, CA, USA) using a SYBR Premix Ex Taq Kit (Takara, Kusatsu, Japan) in accordance with the manufacturer’s instructions. Relative quantification was calculated by the 2^−∆∆CT^ method.

### Statistical analysis

Data were expressed as the mean ± SD of at least three independent experiments and analyzed using GraphPad Prism 5 software (GraphPad Software, La Jolla, CA, USA). Multiple comparisons were performed by one-way analysis of variance (ANOVA) followed by Dunnett’s post hoc test. Differences between two groups were evaluated by Student’s *t* test. Values of *P* < 0.05 were considered statistically significant.

## Results and discussion

### Differentiation of deer tendons and cattle tendons by the LAMP test

**Fig. 2:**
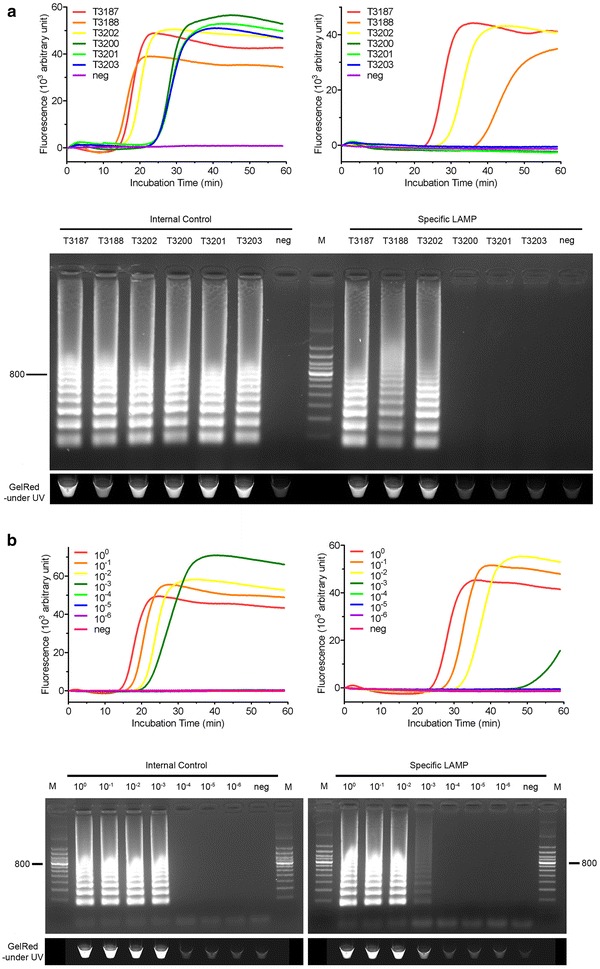
LAMP reaction (**a**) and its sensitivity (**b**). **a** Internal control (*left panel*) and specific LAMP (*right panel*) were real-time monitored by Genie II detector (*upper*), analyzed by gel electrophoresis (*middle*) and visualized under UV light (*bottom*). *Lanes*: M, 100-bp DNA ladder; T3187, T3188, T3202: deer tendon; T3200, T3201, T3203: cattle tendon; neg: negative control. **b** The internal control (*left panel*) and specific LAMP (*right panel*) were performed with serial dilutions of a DNA sample of deer tendon (500 ng/µL). *Lanes* M, 100-bp DNA ladder; 10^0^–10^−6^, amplification with serial diluted DNA; *neg* negative control

The two types of tendons are generally differentiated by size and color. However, as shown in Fig. 1c, fake deer tendons can also be small in size and light in color. To ascertain the animal origins of the tested samples and facilitate on-site application, we developed a LAMP test for the differentiation of deer tendons from cattle tendons. Briefly, internal control and specific LAMP primers were designed to monitor the integrity of the extracted DNA and identify deer tendons, respectively (Table 1). All tendon samples generated amplification products in the internal control reactions, while only deer tendon samples were amplified in the specific LAMP test (Fig. 2a). The amplification results were consistent with the results of DNA barcode sequence analysis. No nonspecific amplification was observed in the negative controls. The results of real-time observation in a fluorescence detector (Fig. 2a, upper) matched the results determined by gel electrophoresis (Fig. 2a, middle). Owing to the large amount of DNA generated in LAMP, positive amplification can be visualized under UV light or by the naked eye when an appropriate dye is added [18, 19]. In the present study, GelRed staining was employed for visualization under UV light (Fig. 2a, bottom). The analytical sensitivity of the developed test was determined by serial dilution of the template DNA. The detection limits for both the internal control reaction and specific LAMP test were 0.5 ng/µL (Fig. 2b). Because the two deer species share highly homologous sequences, it might not be possible to distinguish the two species by DNA sequencing, sequence-based specific PCR [7], or LAMP.

### Effects of tendon extracts on bone remodeling

Bone remodeling involves both osteoblast-induced bone formation and osteoclast-mediated bone resorption. The imbalance of bone remodeling leads to bone loss and osteoporosis [20]. The effects of both types of tendons on bone remodeling in pre-osteoclast Raw264.7 cells and osteoblast-like UMR106 cells were investigated.

**Fig. 3:**
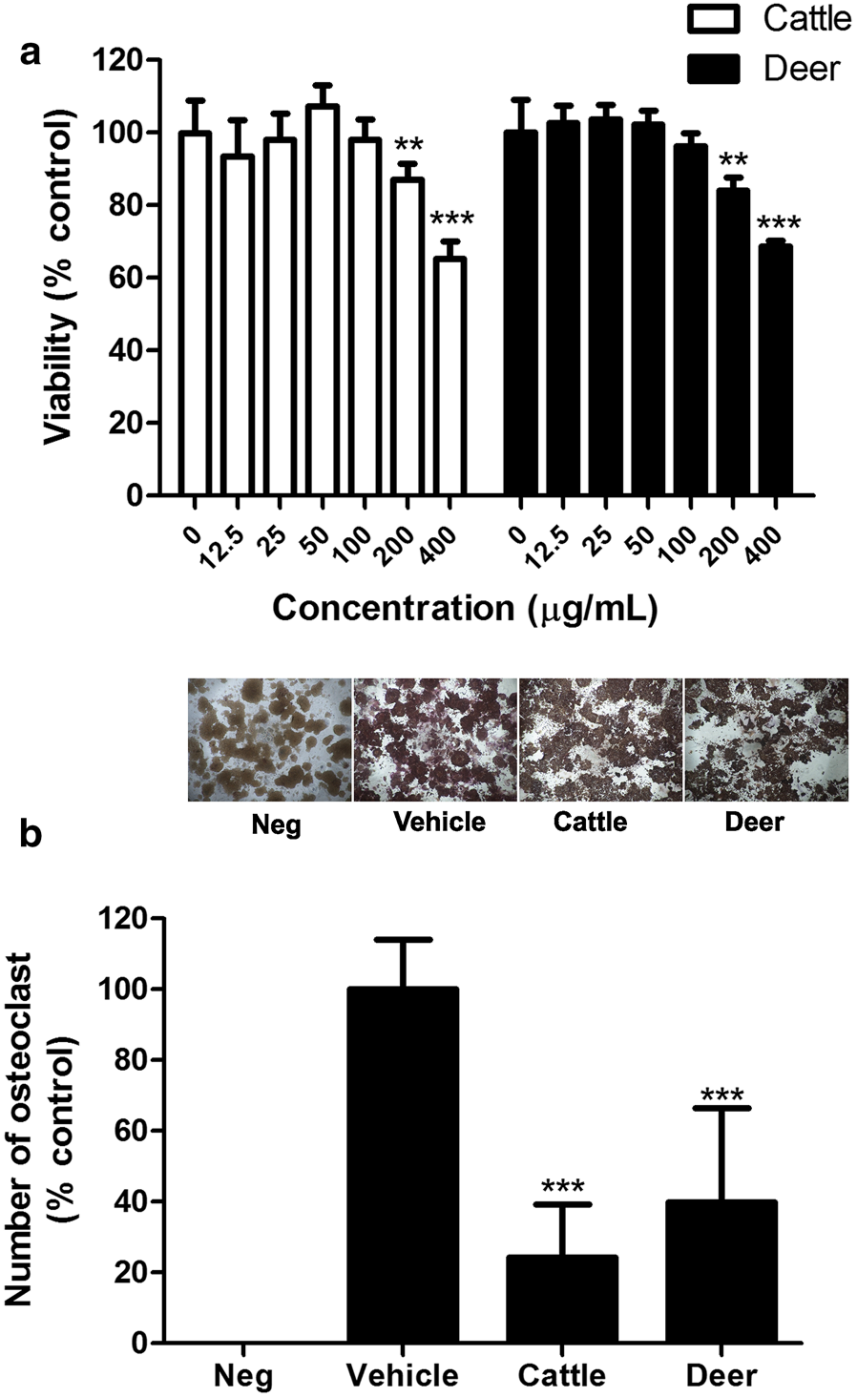
Effects of the tendon extract on cell viability (**a**) and differentiation in RANKL induced-Raw264.7 cells (**b**). **a** Raw 264.7 cells were cultured with different concentration of tendon extracts for 4 days. Cell viability was determined by MTT assay. Results were obtained from three independent experiments (4–6 replicate for each experiment) and expressed as mean ± SD. **P ≤ 0.01, ***P ≤ 0.001 versus the control (“0”). **b** Raw 264.7 cells were cultured with basal medium (Neg), 50 ng/ml RANKL (Vehicle) and 100 µg/mL of each tendon extract for 4 days. A representative photo for TRAP staining (*upper*, magnification: ×40) and the quantitative comparison (*lower*) are shown. Results were obtained from three independent experiments (2–3 replicates for each experiment) and expressed as mean ± SD. ***P ≤ 0.001 versus the vehicle control group

Multinucleated osteoclasts are formed by sequential proliferation, differentiation, and fusion of monocyte or macrophage precursors. The precursor cells are induced to undergo differentiation by RANKL, and the RANKL signaling pathway has been a main target for antiresorptive drugs [21]. Bone resorption occurs in an acidic pH microenvironment, making TRAP an osteoclast marker [22]. In the present study, we first examined the cytotoxic effects of the tendon extracts on Raw264.7 cells. High concentrations of both the deer and cattle tendon extracts (≥200 µg/mL) decreased the number of viable cells on day 4 (Fig. 3a). The cells were then exposed to RANKL stimulation and treated with 100 µg/mL of the tendon extracts. Osteoclasts were characterized as TRAP-positive multinucleated cells. The TRAP staining and number of mature osteoclasts were significantly decreased by both extracts (Fig. 3b). There were no significant differences between the effects of the two types of tendons.

**Fig. 4:**
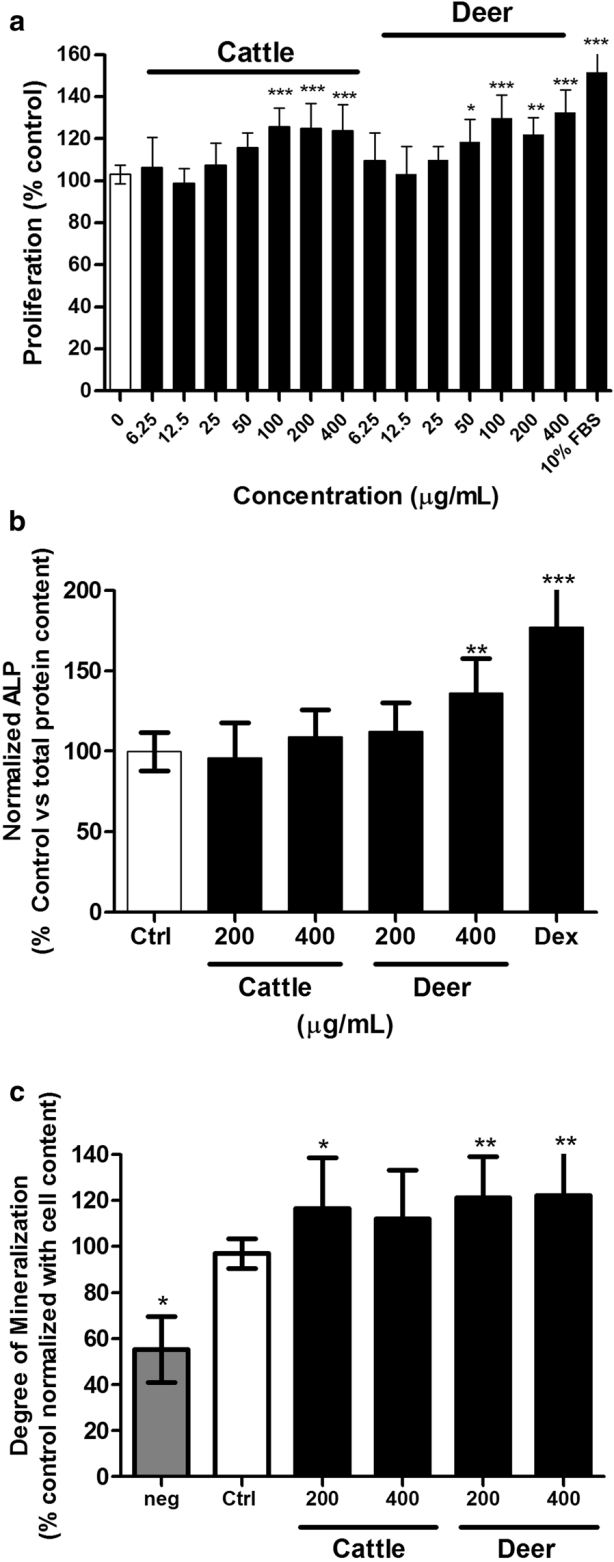
Effect of the tendon extracts on cell proliferation (**a**), alkaline phosphatase activity (**b**) and calcium deposition (**c**) in UMR 106 cells. **a** UMR106 cells were treated with various concentration of tendon extract for 72 h. Cell proliferation was determined by MTT assay. Results were obtained from three independent experiments (3–6 replicates for each experiment) and expressed as mean ± SD. *P ≤ 0.05, **P ≤ 0.01, ***P ≤ 0.001 versus the control group (“0”). **b** UMR106 cells were treated with differentiation medium (Ctrl) and the tendon extract for 5 days. The ALP activity was normalized with the protein content. Cells treated with dexamethasone (Dex) were used as positive control. Results were obtained from three independent experiments (2–4 replicates for each experiment) and expressed as mean ± SD. **P ≤ 0.01, ***P ≤ 0.001 versus the control group (Ctrl). **c** UMR106 cells were treated with basal medium (neg), differentiation medium (Ctrl) and various concentration of tendon extract for 6 days. Calcium deposition were measured by Alizarin red S staining and quantified by a plate reader at 562 nm. Results were obtained from three independent experiments (2–4 replicates for each experiment) and expressed as mean ± SD. *P ≤ 0.05, **P ≤ 0.01 versus the control group (Ctrl)

For bone formation, ALP activity and calcium deposition are markers for evaluating the function of osteoblasts. ALP hydrolyzes pyrophosphate to inorganic phosphate for mineralization [23]. Minerals including calcium and phosphate are deposited in the extracellular matrix during mineralization. The effects of the tendon extracts on the proliferation of UMR106 cells were first determined. Compared with untreated cells, both the deer (50–400 µg/mL) and cattle (100–400 µg/mL) tendon extracts significantly increased the proliferation of UMR106 cells (Fig. 4a). The cells were then induced to undergo differentiation by treatment with β-glycerophosphate and ascorbic acid. The ALP activity was not altered by the cattle tendon extract or 200 µg/mL of the deer tendon extract (Fig. 4b). A higher concentration (400 µg/mL) of the deer tendon extract significantly increased the ALP activity. Compared with the vehicle control, calcium deposition was increased to similar extents by both the deer and cattle tendon extracts (Fig. 4c). However, no significant differences were observed between the same concentrations of the deer and cattle tendon extracts for cell proliferation, ALP activity, or calcium deposition.

The relative gene expressions of bone remodeling-related markers were determined by real-time PCR. Binding of RANKL to its receptor RANK on pre-osteoclasts activates nuclear factor of activated T-cells cytoplasmic 1 (NFATc1) [24] to regulate the expressions of a series of osteoclast-related genes, including those for TRAP and collagenases that destroy the bone matrix such as cathepsin K (CTSK). Inhibition of CTSK has been applied as an antiresorptive strategy [11]. The expressions of NFATc1, TRAP, and CTSK were decreased by both tendon extracts in RANKL-induced Raw264.7 cells (Table 2A). Osteoprotegerin (OPG) and RANKL are cytokines secreted by osteoblasts. RANKL triggers the differentiation of osteoclasts, and this process is inhibited by OPG as a decoy receptor for RANKL [24, 25]. Thus, the expression ratio of OPG/RANKL at a particular location reflects the balance between bone formation and bone resorption [12, 13]. In UMR106 cells, the expression of RANKL was not altered or decreased by the tendon extracts, while the expression of OPG was increased by both the cattle and deer tendon extracts (Table 2B). As a result, the ratio of OPG/RANKL was increased in tendon extract-treated cells compared with control cells. No significant differences were found between the deer and cattle tendon extracts at the same concentrations regarding to the relative expressions of NFATc1, TRAP, and CTSK and the ratio of OPG/RANKL.

**Table 2:** Relative gene expression in Raw 264.7 (A) and UMR106 cells (B)

(A)	NFATc	TRAP	CTSK
Vehicle control	1	1	1
Cattle tendon (100 µg/mL)	0.45 ± 0.17**	0.34 ± 0.11**	0.40 ± 0.18*
Deer tendon (100 µg/mL)	0.74 ± 0.14	0.55 ± 0.20*	0.72 ± 0.18

(B)	OPG	RANKL	OPG/RANKL
Vehicle control	1	1	1
Cattle tendon (200 µg/mL)	1.28 ± 0.82	0.90 ± 0.54	1.37 ± 0.16
Cattle tendon (400 µg/mL)	1.82 ± 0.50	1.18 ± 0.27	1.58 ± 0.49
Deer tendon (200 µg/mL)	1.40 ± 0.76	0.88 ± 0.25	1.56 ± 0.66
Deer tendon (400 µg/mL)	1.72 ± 0.86	0.82 ± 0.20	2.09 ± 0.96*

(A) Raw 264.7 cells were treated with RANKL and 100 µg/mL of tendon extracts for 72 h. (B) UMR 106 cells were treated with differentiation medium together with 200 or 400 µg/mL of both tendon extract for 72 h. For both A and B, the expression of respective genes was determined by real-time PCR and normalized with the housekeeping gene GAPDH. Results were obtained from 4 to 5 independent experiments in duplicate and expressed as mean ± SD

* *P* ≤ 0.05; ** *P* ≤ 0.01 versus vehicle control

The dried mass of tendons consists of 65–80 % collagen (mostly type I collagen) and less than 35 % non-collagenous proteins [26]. Type I collagen is highly conserved in land mammals [27]. The similarity in protein compositions limits the application of protein-based analysis for differentiation between the tendon types. In the present study, the LAMP test could differentiate deer tendons from cattle tendons. Compared with the specific PCR test developed by the Hong Kong Government Laboratory [7], we have introduced an internal control reaction to reduce false-negative results in the specific reaction. Generally, DNA is more or less degraded in dried products, and this leads to negative amplification even in authenticated samples [9]. An internal control reaction to detect both types of tendons can avoid such false-negative results. Besides the conventional gel electrophoresis detection, we also introduced real-time observation and colorimetric detection, which will facilitate on-site detection. Above all, the LAMP reaction is performed at a constant temperature and can be carried out in water kept in a thermal bottle [28]. The isothermal DNA amplification test combined with the fast DNA extraction procedure is easy to adopt by retailers and consumers.

In the present study, both deer tendons and cattle tendons exhibited similar effects in inhibiting the differentiation of Raw264.7 cells into osteoclasts and promoting the cell proliferation and mineralization of UMR106 cells. The collagens from deer tendon and deer antler are effective against osteoporosis in ovariectomized rats [2, 3, 29]. To our knowledge, there have been no reports characterizing the biological effects of cattle tendons either in vivo and in vitro. The results of the present study could not determine whether the beneficial effects of the tendon extracts were caused by the collagenous or non-collagenous fractions. Unlike herbal medicines that have standard protocols for their partition and isolation, crude tendon extracts are mixtures of many proteins and other molecules. We have started to characterize the effective components in the tendon extracts and to compare the beneficial effects of the two types of tendon extracts in ovariectomized rat models.

## Conclusion

This study demonstrated that the developed isothermal LAMP test can distinguish between deer tendons and cattle tendons. Biological assays showed that both types of tendons exhibited similar beneficial effects on bone remodeling.

## Additional file

10.1186/s13020-015-0065-6 **Table S1.** Information for the tested samples. **Table S2.** Primer sequences for real-time PCR.
