# Supplementary material for: Differentiation of deer tendons from cattle tendons by a loop-mediated isothermal amplification (LAMP) test and bone remodeling bioassays
Source: Chin Med. 2015 Nov 12;10:33. doi: 10.1186/s13020-015-0065-6 (PMC4642741; doi:10.1186/s13020-015-0065-6)
Supplement: Supplementary file 1 — 10.1186/s13020-015-0065-6 Table S1. Information for the tested samples. Table S2. Primer sequences for real-time PCR. [file 13020_2015_65_MOESM1_ESM.docx]

| Sample code | Source | Claimed identity | Identity found |
| --- | --- | --- | --- |
| T3187 | Guangzhou, China | Deer tendon | *Cervus sp.* |
| T3188 | Guangzhou, China | Deer tendon | *Cervus sp.* |
| T3202 | Hong Kong, China | Deer tendon | *Cervus sp.* |
| T3200 | Hong Kong, China | Cattle tendon | *Bos taurus* |
| T3201 | Hong Kong, China | Cattle tendon | *Bos taurus* |
| T3203 | Guangzhou, China | Deer tendon | *Bos taurus* |

Table S1 Information for the tested samples

| Gene | Forward Primer 5’-3’ | Reverse Primer 5’-3’ |
| --- | --- | --- |
| OPG | GACGAGATTGAGAGAACGAG | GGTGCTTGACTTTCTAGGTG |
| RANKL | TCAGGAGTTCCAGCTATGAT | CCATCAGCTGAAGATAGTCC |
| GAPDH(rat) | TGAGGTGACCGCATCTTCTTG | TGGTAACCAGGCGTCCGATA |
| TRAP | ACACAGTGATGCTGTGTGGCAACTC | CCAGAGGCTTCCACATATATGATGG |
| Cathepsin K | GGCCAACTCAAGAAGAAAAC | GTGCTTGCTTCCCTTCTGG |
| NFATc1 | GGGTCAGTGTGACCGAAGAT | GGAAGTCAGAAGTGGGTGGA |
| GAPDH(mouse) | AACTTTGGCATTGTGGAAGG | ACACATTGGGGGTAGGAACA |

Table S2 Primer sequences for real-time PCR
